# Sample size determination for point-of-care COVID-19 diagnostic tests: a Bayesian approach

**DOI:** 10.1186/s41512-023-00153-1

**Published:** 2023-08-18

**Authors:** S. Faye Williamson, Cameron J. Williams, B. Clare Lendrem, Kevin J. Wilson

**Affiliations:** 1 Biostatistics Research Group, Population Health Sciences Institute, Newcastle University, Newcastle upon Tyne, UK; 2 NIHR Newcastle In Vitro Diagnostic Cooperative, Newcastle University, Newcastle upon Tyne, UK; 3 School of Mathematics, Statistics and Physics, Newcastle University, Newcastle upon Tyne, UK

**Keywords:** Bayesian assurance, COVID-19, Diagnostic accuracy study, Precision, Sample size, Sensitivity, Specificity

## Abstract

**Background:**

In a pandemic setting, it is critical to evaluate and deploy accurate diagnostic tests rapidly. This relies heavily on the sample size chosen to assess the test accuracy (e.g. sensitivity and specificity) during the diagnostic accuracy study. Too small a sample size will lead to imprecise estimates of the accuracy measures, whereas too large a sample size may delay the development process unnecessarily. This study considers use of a Bayesian method to guide sample size determination for diagnostic accuracy studies, with application to COVID-19 rapid viral detection tests. Specifically, we investigate whether utilising existing information (e.g. from preceding laboratory studies) within a Bayesian framework can reduce the required sample size, whilst maintaining test accuracy to the desired precision.

**Methods:**

The method presented is based on the Bayesian concept of assurance which, in this context, represents the unconditional probability that a diagnostic accuracy study yields sensitivity and/or specificity intervals with the desired precision. We conduct a simulation study to evaluate the performance of this approach in a variety of COVID-19 settings, and compare it to commonly used power-based methods. An accompanying interactive web application is available, which can be used by researchers to perform the sample size calculations.

**Results:**

Results show that the Bayesian assurance method can reduce the required sample size for COVID-19 diagnostic accuracy studies, compared to standard methods, by making better use of laboratory data, without loss of performance. Increasing the size of the laboratory study can further reduce the required sample size in the diagnostic accuracy study.

**Conclusions:**

The method considered in this paper is an important advancement for increasing the efficiency of the evidence development pathway. It has highlighted that the trade-off between lab study sample size and diagnostic accuracy study sample size should be carefully considered, since establishing an adequate lab sample size can bring longer-term gains. Although emphasis is on its use in the COVID-19 pandemic setting, where we envisage it will have the most impact, it can be usefully applied in other clinical areas.

## Background

In response to the ongoing and continually evolving COVID-19 pandemic, early detection of infectious individuals is critical to successful outbreak containment, and thus there is a need to evaluate and deploy accurate point-of-care (PoC) diagnostic tests rapidly [19, 28]. The development of new diagnostic tests ideally consists of various stages, including an analytical validity (laboratory) study, a diagnostic accuracy study, and a clinical utility study [15]. Diagnostic accuracy studies compare the results of the index test against those obtained from the best available reference standard to evaluate its ability to correctly identify patients with and without the target condition. Diagnostic accuracy is typically measured by the clinical sensitivity and specificity, which should ideally attain pre-defined minimum levels to be considered clinically useful and be estimated with sufficient precision. Whether the required precision is attained is affected by the sample size used in the diagnostic accuracy study, which is often difficult to choose [5]. Too small a sample size may lead to imprecise estimates of accuracy measures, whereas too large a sample size will yield greater precision but may contribute to longer recruitment times (especially when prevalence is low), require greater resources and delay the development process unnecessarily.

Target product profiles (TPPs) are often used to inform sample size calculations in diagnostic accuracy studies. TPPs outline the required profiles of a target product, including the minimally acceptable and desirable sensitivities and specificities, based on the opinion of healthcare professionals and scientists. These values are subject to review and may be updated as knowledge of the disease and clinical needs change. For PoC SARS-CoV-2 viral detection tests, which are the focus of this paper, the TPPs published by the UK’s Medicines and Healthcare products Regulatory Agency (MHRA) [22] on June 15, 2020, are provided in Table 1. PoC tests refer to in vitro diagnostic tests intended to be used by a healthcare professional outside of a laboratory in primary or secondary care environments, or other settings such as schools or a person’s home. They generally provide results much quicker than laboratory-based tests.

During the COVID-19 pandemic, national and international regulatory agencies produced conflicting minimum TPPs and, based on these, recommended a range of sample sizes to use when developing new tests. For example, as shown in Table 1, the UK’s MHRA [22] set the minimum desirable sensitivity to be achieved at the clinical performance stage to be $97\%$. The World Health Organisation [39] stipulated that the minimum desirable sensitivity should be $90\%$. Sammut-Powell et al. [32] illustrate the impact of using these different sample sizes on the expected performance of COVID-19 diagnostic tests in practice, by evaluating the probability that they will fail to meet target specifications after implementation.

Standard practice for determining sample sizes in diagnostic accuracy studies is based on hypothesis testing or equivalent confidence intervals and associated power calculations. If the aim is to attain a required precision around estimates of diagnostic accuracy, choosing a sample size which yields confidence intervals (CIs) of the target width may be more appropriate [29]. For a review of procedures used to determine sample sizes in diagnostic accuracy studies, refer to [21, 40].

Most diagnostic accuracy studies do not perform and report sample size calculations [20, 33]. For example, in the survey by Bachmann et al. [2], only 5% of 43 diagnostic accuracy studies published in eight leading medical journals reported sample size calculations. Similarly, in Ochodo et al. [24], only 11% of 126 published diagnostic accuracy studies included a sample size calculation. More recently, a survey of 89 diagnostic accuracy studies for depression screening tools revealed that only 8% mentioned a sample size calculation and the number of patients in most studies was too small to provide precise estimates [34]. However, sample size determination is increasingly requested by regulatory authorities and the updated Standards for Reporting of Diagnostic Accuracy Studies (STARD) guideline [3, 10] specifically states that “the intended sample size and how it was determined” should be reported for diagnostic accuracy studies. Despite this, compliance is only moderate and, as highlighted in [31], “methodological improvements are needed to guide considerations of sample size in diagnostic research”, which we aim to contribute to in this paper.

We consider sample size determination from a Bayesian perspective by applying the *Bayesian assurance method* (BAM), proposed in [37] and outlined in the “Bayesian assurance method (BAM)” section, to diagnostic accuracy studies for PoC SARS-CoV-2 viral detection tests. In contrast to traditional power, which represents a conditional probability that the study is a “success” given the values chosen for the unknown design parameter(s), assurance is an *unconditional* probability which incorporates parameter uncertainty by averaging the power over the parameter range [25]. Conceptually, the assurance can therefore be viewed as an expected power which offers a robust alternative to standard power. In this paper, the assurance represents the unconditional probability of obtaining precise sensitivity and/or specificity estimates (based on a target interval width) at the end of the diagnostic accuracy study. We explore the effect of utilising information from an earlier laboratory study to calculate the sample size required to attain the desired assurance level. This has the potential to reduce required sample sizes, when compared to those obtained using traditional power-based approaches (see the “Simulation structure” section), and thus accelerate the evidence development pathway, which is especially important for pandemic management. As different variants of COVID-19 emerge, it is even more pressing to be able to adapt and re-assess the diagnostic properties of tests on variants of concern quickly, which may require an updated sample size.

The sensitivity and specificity for a particular population may not be generalisable to different populations or settings where severity of symptoms differ [22]. For example, testing populations with more severe COVID-19 symptoms where viral loads are likely to be higher, such as in intensive care, will give rise to higher sensitivity. Testing in general practice or schools, where viral loads are lower or more people are asymptomatic, will increase the risk of false negatives and reduce the sensitivity of the test (e.g. 12, 38). Other examples affecting the accuracy estimates between populations include if the quality of the sample varies, which may depend on who administers the test (e.g. health-care professional vs. self-testing), or if the virus has mutated and the test does not detect the new variant. These issues have been raised by the Royal Statistical Society [30] who were concerned that COVID-19 antigen tests had come to market without adequate statistical evaluation of their performance for many of their subsequent uses [17, 27]. The COVID-19 pandemic therefore highlights the importance of conducting rigorous and unbiased evaluations of tests in a variety of settings to ensure tests produce accurate and precise estimates in their intended clinical setting [13]. Accordingly, in the “Results” section, we assess the sample sizes required in different real world settings (e.g. schools, emergency departments (EDs), general practice) and different time points during pandemic waves.

Assurance remains a relatively new concept to many biostatisticians and regulators, and software implementation for public use has been identified as an unmet practical need [8]. Therefore, to complement this paper, we have developed a publicly available interactive R Shiny application, which can be used by diagnostic test developers and researchers to perform sample size calculations using the BAM. The link to this is provided in the “Software implementation” section.

**Table 1:** Target product profiles (TPPs) for SARS-CoV-2 viral detection tests from the MHRA [22]

TPP	Sensitivity (95% CI)	Specificity (95% CI)
Acceptable	80% (70–100%)	95% (90–100%)
Desirable	97% (93–100%)	99% (97–100%)

## Methods

In this section, we describe how to obtain sample sizes for diagnostic accuracy studies using Bayesian assurance and commonly used frequentist methods. In both cases, we aim to ensure that the sensitivity and/or specificity of the test is estimated to a chosen degree of precision. We measure precision via the width of the corresponding interval estimate(s) following the diagnostic accuracy study.

### Bayesian assurance method (BAM)

Assurance can be described as the unconditional probability that a study is “successful”. A successful diagnostic accuracy study will result in precise sensitivity and/or specificity estimates. Therefore, in this context, assurance represents the probability that, for a chosen sample size, the resulting interval estimate(s) will have width(s) narrower than some pre-specified target(s), without conditioning on point estimates of the sensitivity, specificity and prevalence, as would be necessary in a power calculation. We use interval estimates in the form of Bayesian credible intervals for the sensitivity and/or specificity. The sample size can then be chosen as the smallest value which provides the desired level of assurance (typically 80% or 90%). This approach is known as the *Bayesian assurance method* (BAM), and full details are provided in [37]. In the following paragraphs, we outline the main elements of the BAM.

Suppose we are interested in assuring the precision of the *sensitivity* $\lambda$ following the diagnostic accuracy study, by targeting some desirable width of the corresponding interval. Conditional on the true number of individuals in the study with the target disease, the number who obtain a positive test result is binomially distributed with probability of success given by the sensitivity. If the prior distribution for the sensitivity is taken to be a beta distribution (with parameters $a_\lambda$ and $b_\lambda$, say) then the analysis is conjugate. This means that the posterior distribution for the sensitivity is also a beta distribution with updated parameters given by $a_\lambda + n_{11}$ and $b_\lambda + n_{21}$, where $n_{11}$ and $n_{21}$ represent the number of true positives and false negatives, respectively. The relevant quantiles of this beta distribution form the limits of the posterior credible interval. If we know the number of individuals with the target disease and the true sensitivity of the test, we can evaluate whether the interval meets the target width for each combination of positive/negative test results, as well as the probability of observing the corresponding combination of test results. From this, we can calculate the probability that the width of the credible interval meets the target.

In practice, however, the number of individuals with the target disease and the true sensitivity of the test will be unknown. Therefore, we take the expectation with respect to the prior distributions on the sensitivity and the number of individuals in the study who have the target disease. The latter term depends on the prevalence and, consequently, we also need to integrate over the possible prevalence values in the target population. If the prior distribution on the prevalence is also a beta distribution, the resulting assurance can be written in closed form (see [37], Eq. 2).

If the target accuracy measure is specificity, the assurance is evaluated analogously. The BAM can also be used to assure the sensitivity and specificity together, rather than separately. In this case, we average over the priors for both quantities. If they are assigned independent beta prior distributions, the assurance can still be expressed in closed form (see [37], Eq. 3). Otherwise, we revert to simulation and numerical integration methods. Note that this does not provide the same result as either of the sample size calculations considering sensitivity or specificity independently. This contrasts with common practice where, for simplicity, the larger of the sample sizes from the two separate power calculations is often taken when testing both sensitivity and specificity together [40]. However, this does not necessarily provide the required power for both together.

The prior distributions chosen for the sensitivity, specificity and prevalence in the diagnostic accuracy study may be non-informative or elicited from experts. Elicited distributions can include opinions from multiple experts or be combined with data from other sources [35]. However, in the development of diagnostic tests and, in particular, rapid COVID-19 detection tests, data will be available from previous developmental stages of the test (typically, the analytic validity stage). If this data is from the same target population, then we can use the posterior distributions on the measures of interest from the analytic validity stage as the prior distributions to choose the sample size for the diagnostic accuracy stage. Again, if independent beta priors are chosen before the analytic validity stage, the analysis is conjugate and we will have independent beta priors for the diagnostic accuracy study.

### Simulation structure

The simulation for the BAM takes the following steps:

1. Choose values for the sensitivity $\lambda _T$ and/or specificity $\theta _T$ of a rapid COVID test which are consistent with the acceptable ($80\%$ sensitivity and $95\%$ specificity) or desirable ($97\%$ sensitivity and $99\%$ specificity) TPPs. Let $\rho _T$ denote the “true” prevalence in the target population, which can be chosen to reflect different settings of interest. Specify the target width(s) for the interval(s) and the desired assurance level (typically 80% or 90%).
2. Set the prior distributions for the sensitivity, specificity and prevalence to be (independently)
  $$\begin{aligned} \lambda\sim & {} \text {Beta}(a_\lambda , b_\lambda ), \\ \theta\sim & {} \text {Beta}(a_\theta , b_\theta ), \\ \rho\sim & {} \text {Beta}(a_\rho , b_\rho ). \end{aligned}$$
  In each case, *a* and *b* are chosen to represent beliefs prior to the analytic validity phase ($a=b=1$ would give a flat prior).
3. For $i=1,\ldots ,I$ (where *I* is the total number of iterations), sample the lab results from the analytic validity phase
  $$\begin{aligned} n_{11}^{(i)}\mid \lambda _T\sim & {} \text {Bin}(n_c,\lambda _T), \\ n_{22}^{(i)}\mid \theta _T\sim & {} \text {Bin}(n_{\bar{c}},\theta _T), \end{aligned}$$
  where $n_{11}$ ($n_{22}$) is the number of true positives (negatives) and $n_c$ ($n_{\bar{c}}$) is the number of individuals in the sample with (without) COVID-19, as determined by the reference standard test; see Table 2(i).
4. Combine the priors from step 2 with the data from the analytic validity phase in step 3 to form the prior distributions for the diagnostic accuracy study. That is, omitting the conditioning,
  $$\begin{aligned} \lambda\sim & {} \text {Beta}(a_\lambda + n_{11}^{(i)}, b_\lambda + n_{21}^{(i)}), \\ \theta\sim & {} \text {Beta}(a_\theta + n_{22}^{(i)}, b_\theta + n_{12}^{(i)}). \end{aligned}$$
5. Using the equations provided in [37], calculate the assurance for a range of sample sizes $N^{(i)} = N_0^{(i)}, N_0^{(i)}+1, N_0^{(i)}+2, \ldots$, where $N_0^{(i)}$ is the initial sample size at which we begin the search.
6. The minimum sample size $N_{min}^{(i)}$ which gives rise to the desired assurance (typically, 80% or 90%) is chosen as the sample size to use in the diagnostic accuracy study.
7. Simulate data from the prospective diagnostic accuracy study
  $$\begin{aligned} m_{c}^{(i)}\mid \rho _T\sim & {} \text {Bin}(N_{min}^{(i)},\rho _T), \\ m_{11}^{(i)}\mid \lambda _T\sim & {} \text {Bin}(m_c^{(i)},\lambda _T), \\ m_{22}^{(i)}\mid \theta _T\sim & {} \text {Bin}(m_{\bar{c}}^{(i)},\theta _T), \end{aligned}$$
  where $m_{\bar{c}}^{(i)}=N_{min}^{(i)}-m_c^{(i)}$ represents the number of individuals without COVID in the diagnostic accuracy study; see Table 2(ii).
8. Using the data from step 7, update the posterior distributions of $\lambda ,\;\theta ,\;\rho$
  $$\begin{aligned} \lambda \mid m_{11}^{(i)}\sim & {} \text {Beta}(a_\lambda +n_{11}^{(i)}+m_{11}^{(i)},b_\lambda +n_{21}^{(i)}+m_{21}^{(i)}), \\ \theta \mid m_{22}^{(i)}\sim & {} \text {Beta}(a_\theta +n_{22}^{(i)}+m_{22}^{(i)},b_\theta +n_{12}^{(i)}+m_{12}^{(i)}), \\ \rho \mid m_{c}^{(i)}\sim & {} \text {Beta}(a_\rho +m_c^{(i)},b_\rho +m_{\bar{c}}^{(i)}). \end{aligned}$$
  Use the posterior measures to determine if the study met the success criteria, i.e. is the posterior credible interval width for the accuracy measure(s) smaller than the target width (based on the TPPs)?
9. Return to step 3.

After *I* iterations, we can evaluate the different sample sizes required and the proportion of times the diagnostic accuracy study was a success.

**Table 2:** (i) A $2\times 2$ contingency table for the COVID-19 analytic validity study. (ii) A $2\times 2$ contingency table for the prospective COVID-19 diagnostic accuracy study

(i)	COVID-19	No COVID-19	Total
Positive	$n_{11}$	$n_{12}$	
Negative	$n_{21}$	$n_{22}$	
Total	$n_c$	$n_{\bar{c}}$	$n_c+n_{\bar{c}}$
(ii)	COVID-19	No COVID-19	Total
Positive	$m_{11}$	$m_{12}$	
Negative	$m_{21}$	$m_{22}$	
Total	$m_c$	$m_{\bar{c}}$	$N_{min}$

#### Illustrative example

We concretise the above steps using a specific example to illustrate how the sample size for a diagnostic accuracy study can be chosen using assurance.

Consider a setting in which $\lambda _T = 0.8$, $\theta _T = 0.95$ and $\rho _T = 0.1$. Suppose we assign the following prior distributions to the unknown parameters before the lab study

$$\begin{aligned} \lambda\sim & {} \text {Beta}(1,1), \\ \theta\sim & {} \text {Beta}(1,1), \\ \rho\sim & {} \text {Beta}(13.56, 122.06), \end{aligned}$$

where the parameters for the prevalence prior have been chosen such that the prior mean for the prevalence is 0.1 and the 95% interval width is 0.1.

Suppose we observe the following data from the lab study (Table 3(i)), where we have 30 COVID patients and 30 non-COVID patients (as determined by the reference standard test).

**Table 3:** (i) Example results from the COVID-19 analytic validity study. (ii) Example results from the prospective COVID-19 diagnostic accuracy study

(i)	COVID-19	No COVID-19	Total
Positive	24	1	25
Negative	6	29	35
Total	30	30	60
(ii)	COVID-19	No COVID-19	Total
Positive	25	9	34
Negative	7	280	287
Total	32	289	321

**Fig. 1:**
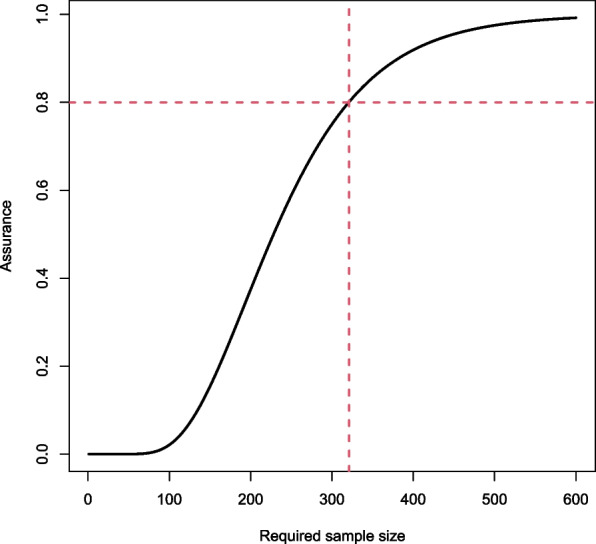
Assurance curve. The dashed horizontal line represents the target assurance level

Combining these lab results with the above prior distributions via Bayes’ theorem gives the following updated distributions for the sensitivity and specificity (omitting the conditioning)

$$\begin{aligned} \lambda\sim & {} \text {Beta}(1 + 24, 1 + 6), \\ \theta\sim & {} \text {Beta}(1 + 29, 1 + 1), \end{aligned}$$

which form the prior distributions for the diagnostic accuracy study. We can then calculate the assurance for a range of sample sizes and choose the minimum sample size which yields the target assurance level. For this example, the required sample size is 321 when the target assurance is 80%, as illustrated by the corresponding assurance curve in Fig. 1. This means that by using a sample size of 321 in the diagnostic accuracy study, the resulting sensitivity and specificity intervals will have sufficient precision with probability 80%.

Suppose the diagnostic accuracy study provides the results in Table 3(ii), then the posterior distributions are updated as follows (omitting the conditioning)

$$\begin{aligned} \lambda\sim & {} \text {Beta}(25 + 25,\; 7 + 7), \\ \theta\sim & {} \text {Beta}(30 + 280,\; 2 + 9), \\ \rho\sim & {} \text {Beta}(13.56 + 32,\; 122.06 + 289), \end{aligned}$$

and can be used to obtain the relevant posterior summary measures.

### Alternative methods

In this section, we outline alternative methods that are used in practice to determine sample sizes for diagnostic accuracy studies with binary outcomes. Since we are interested in estimating the sensitivity and/or specificity of a test with sufficient precision, it is important to consider the widths of the corresponding confidence intervals. To construct confidence intervals for the sensitivity and specificity, we will use the following standard intervals for binomial proportions. These have been discussed extensively in the literature [14].

1. The *Wald interval* [40] is the most well-known confidence interval for proportions and is based on the asymptotic normal approximation to the binomial distribution. For sensitivity, it takes the form $\hat{\lambda } \pm z_{\alpha } \sqrt{\frac{\hat{\lambda } ( 1 - \hat{\lambda } )}{m_c}}$, where $z_{\alpha }$ is the $1-\alpha$ quantile of the standard normal distribution and $\hat{\lambda }=m_{11}/m_c$ is the maximum likelihood estimate of sensitivity (with $m_{11}$ the number of true positives and $m_c$ the total number of COVID patients). The Wald interval is criticised for having a low coverage probability, i.e. the percentage of times that the interval includes the true sensitivity is typically much smaller than desired.
2. The Clopper-Pearson *(CP) interval* [9] is based on the exact binomial distribution (so is sometimes referred to as the ‘exact’ method). Although the coverage is considerably higher than the Wald interval, the CP interval is very conservative and produces wider intervals than necessary.
3. The Agresti-Coull *(AC) interval* [1] is a slightly modified version of the Wald interval to improve coverage, giving rise to an asymmetric interval no longer centred on $\hat{\lambda }$.
4. The Wilson *(or score) interval* [36] is another modification of the normal approximation, which is centred on the same value as that used in the AC interval.
5. The *Jeffreys interval* [4] uses a Bayesian approach which assumes that the unknown binomial proportion has a non-informative Beta(1/2, 1/2) prior distribution (referred to as Jeffreys prior).

Each of these intervals require specification of: the expected sensitivity and/or specificity; the required precision of the sensitivity and/or specificity estimates, i.e. the target interval width; the significance level $\alpha$ and the target power.

We will compute the above intervals using the BinomCI function from the DescTools R package. By simulating these intervals repeatedly (10,000 times), we choose the minimum sample size which gives rise to the desired proportion of intervals (equal to the target power) smaller than the target width.

## Simulation study: application to COVID-19

We implement the BAM and alternative methods via simulation in a variety of scenarios motivated by the COVID-19 pandemic setting. This section provides details of the simulation study conducted.

We focus on assuring the half width of the one-sided $100(1 - \alpha )\%$ posterior credible interval(s) for the accuracy measure(s), where $\alpha =0.05$ is the significance level. The target interval half widths for the sensitivity $w^*_\lambda$ and/or specificity $w^*_\theta$ of SARS-CoV-2 viral detection tests are displayed in Table 4. These are calculated as the distance between the relevant TPP and the lower limit of the corresponding 95% interval (shown in Table 1). From the illustrative example above (in the “Illustrative example” section), we obtain posterior medians and 95% one-sided credible intervals of 0.784 (0.692, 1) and 0.967 (0.948, 1) for sensitivity and specificity, respectively. The corresponding one-sided interval half widths, 0.092 and 0.019, are given by the distance between the posterior medians and the lower limits of the interval. Since these are smaller than their respective acceptable target widths of 0.10 and 0.05, the diagnostic accuracy study can be considered a success according to our definition.

**Table 4:** Target interval half widths $w^*$ for sensitivity $\lambda$ and specificity $\theta$ of SARS-CoV-2 viral detection tests

TPP	$w^*_\lambda$	$w^*_\theta$
Acceptable	0.10	0.05
Desirable	0.04	0.02

In order to create a comprehensive picture of the performance of the BAM in a variety of COVID settings, we run simulations for a range of parameter combinations. In particular, we vary:

1. Prevalence of COVID in the target population: $\rho _T = (0.05, 0.10, 0.20, 0.30)$. These values reflect fluctuations in the prevalence of COVID across different real-life settings, patient groups, locations and during the course of the pandemic (e.g. [6, 11, 26]). Prevalences were generally low in the community but high in secondary care settings, particularly the ED, and very high in areas where tests were being used to confirm positivity.
2. Number with, $n_c$, and without, $n_{\bar{c}}$, COVID in the initial lab study: $n_c=n_{\bar{c}}=(10,20,30,40,50)$, to give total lab study sample sizes of $n_0^T=(20,40,60,80,100)$.
3. Target assurance: we consider a target assurance of 80%, which is often used in practice, and 90%, which is the ideal value in the COVID context.

When implementing the BAM, we set the initial sample size $N_0^{(i)}$ to 10 and increase by one thereafter (step 5 in the “Simulation structure” section) until the target assurance is attained. We set the beta prior distribution parameters before the lab study to reflect a lack of knowledge (i.e. $a=b=1$). We simulate $I=10,000$ replications for each of the scenarios listed above and summarise the proportion of times that the posterior credible interval widths for the sensitivity and/or specificity are within the target width, i.e. attain the desired precision. This provides an estimate of the probability that the diagnostic accuracy study is successful.

**Fig. 2:**
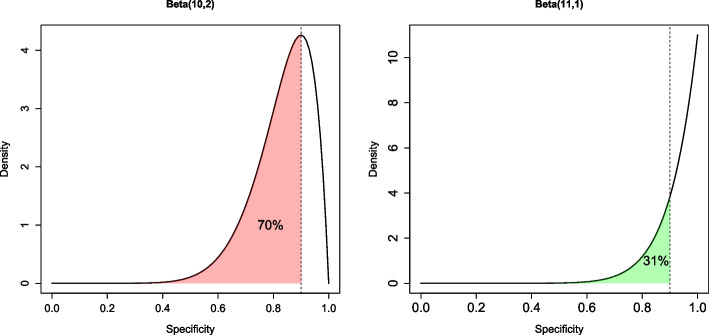
Examples of updated prior distributions for the specificity following a “pessimistic” (left) and “optimistic” (right) lab study. The dashed vertical line represents the acceptable specificity target

In practice, if the results from the lab study indicate that the performance of the diagnostic test is unsatisfactory, then the test should not proceed to the next stage of development, to avoid wasting resources. Therefore, for each *i*, if the probability of the sensitivity and/or specificity being below the corresponding target is above $50\%$, based on the updated prior distributions following the lab study (step 4 in the “Simulation structure” section), this data set is discarded and does not contribute to the sample size calculation for the diagnostic accuracy study. For example, suppose we are interested in the specificity of the test, which we assume follows a Beta(1, 1) flat prior distribution before the lab study. If the true number of participants without COVID in the lab study is $n_{\bar{c}}=10$; $n_{22}=9$ of which correctly test negative and $n_{12}=1$ which incorrectly tests positive, then the distribution on the specificity is updated to Beta(1+9, 1+1) (left plot in Fig. 2). The probability of the specificity lying below the acceptable target of 0.9 is $70\%$ (red shaded region in Fig. 2). Since this is greater than $50\%$, we consider this a ‘pessimistic’ lab result and exclude it from the sample size calculation. However, if the lab results appear promising, we proceed to obtain the sample size for which the posterior probability of the interval being sufficiently narrow meets the desired target assurance. For example, if all ten patients in the lab study correctly test negative, so that the specificity follows a Beta(11, 1) distribution, the probability that the specificity is below the target of 0.9 is $31\%$ (green shaded region in Fig. 2) and hence this lab result will contribute to the sample size calculation for the diagnostic accuracy study.

## Results

In this section, we present the results corresponding to the acceptable TPPs presented in Table 1. Results for the desirable TPPs (with the pessimistic data included) are provided in Appendix “Performance of the BAM for the desirable TPPs” and yield similar conclusions.

### Bayesian assurance method (BAM)

We first focus on the sample sizes obtained from the BAM for a variety of scenarios, before comparing them to those obtained via the alternative methods outlined in the “Alternative methods” section.

**Fig. 3:**
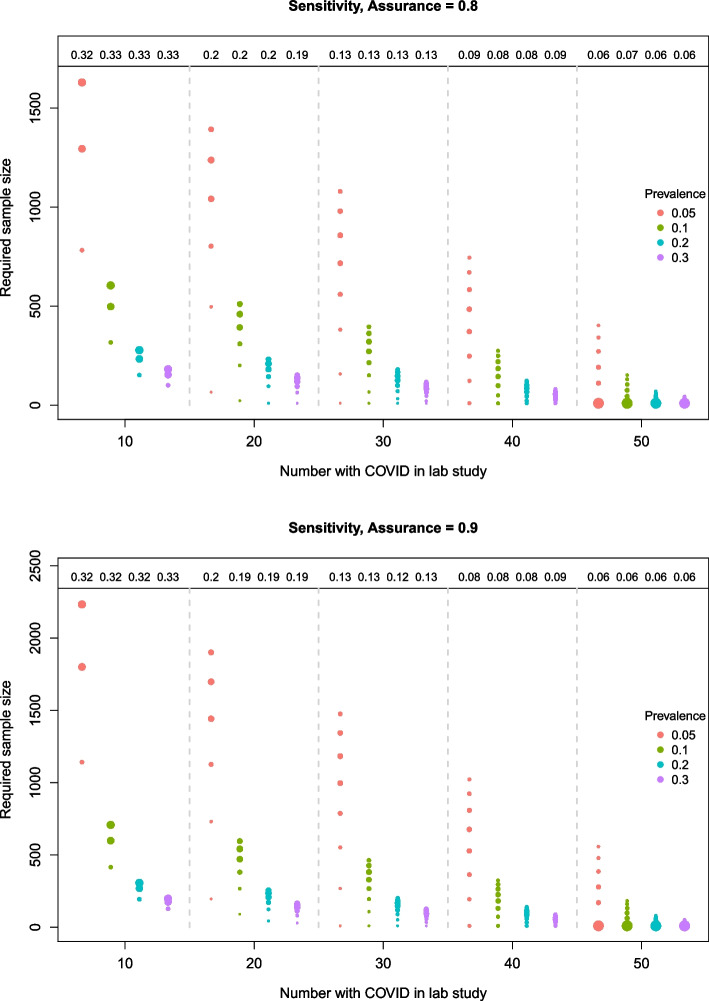
Required sample sizes for different lab sample sizes and prevalences based on the BAM for *sensitivity*. The top and bottom plots correspond to a target assurance of 0.8 and 0.9, respectively

**Fig. 4:**
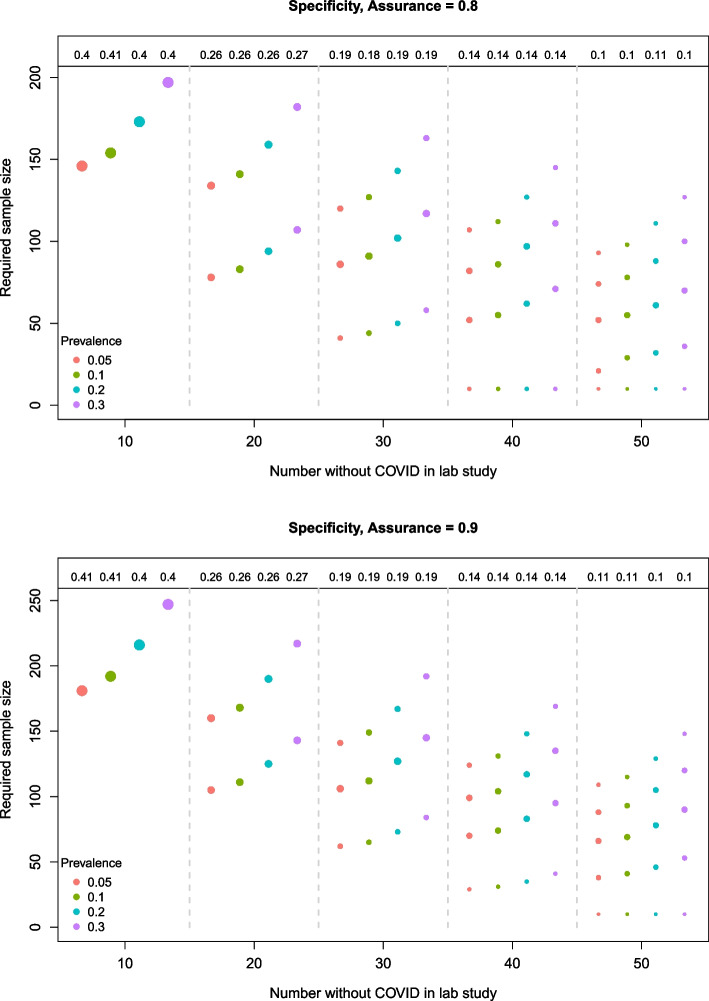
Required sample sizes for different lab sample sizes and prevalences based on the BAM for *specificity*. The top and bottom plots correspond to a target assurance of 0.8 and 0.9, respectively

**Fig. 5:**
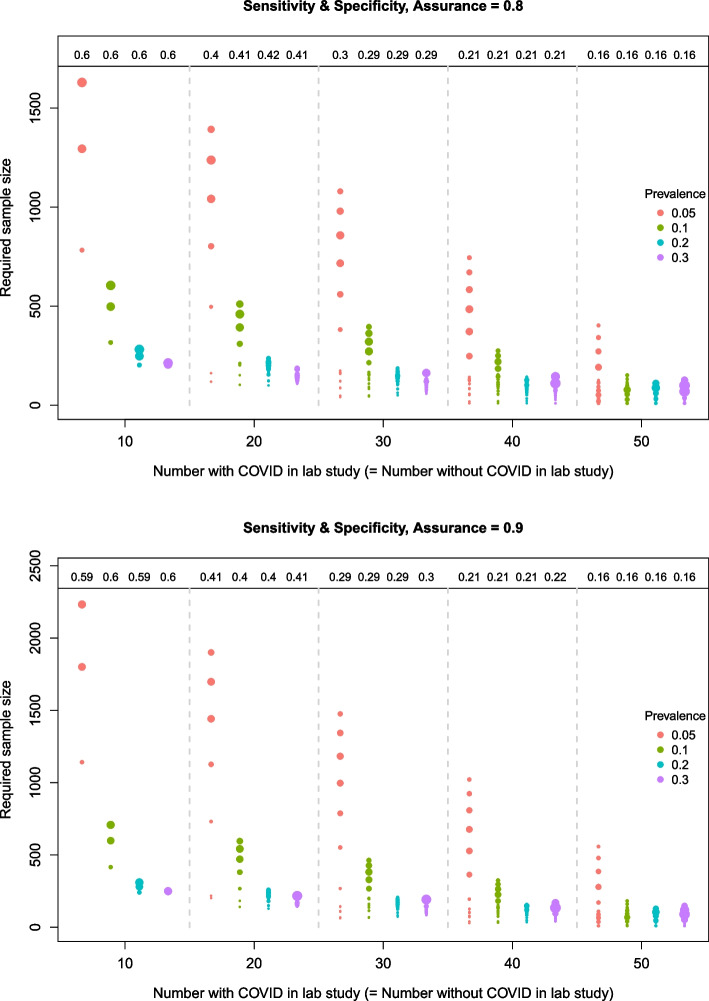
Required sample sizes for different lab sample sizes and prevalences based on the BAM for *sensitivity and specificity* together. The top and bottom plots correspond to a target assurance of 0.8 and 0.9, respectively

Figures 3, 4 and 5 illustrate how the sample size required for the diagnostic accuracy study to attain the desired assurance level varies with the number of COVID cases in the lab study and the prevalence of COVID in the target population. The number of circles represents the number of unique sample sizes that were obtained in the simulation study; each one corresponding to a different set of lab results. The size of the circles is proportional to the number of times the corresponding sample size occurred in the $I=10,000$ simulations. For example, when $n_c=10$ in the top plot of Fig. 3 (i.e. sensitivity, target assurance of 0.8), there are three possible sample sizes corresponding to lab results in which the number of true positives is $n_{11}=8$, 9 and 10. For a prevalence of 0.05, the sample sizes are 1630, 1295 and 783, which occur in approximately $30\%$, $27\%$ and $11\%$ of the *I* simulations, respectively. For a larger prevalence of 0.3, the sample sizes are 182, 154 and 101, which similarly occur in approximately $30\%$, $27\%$ and $11\%$ of the simulations. Note that these are simply the binomial probabilities of obtaining $n_{11}$ true positives out of $n_c$ COVID patients (see step 3 in the “Simulation structure” section). The remaining $32\%$ of the simulated samples were discarded due to pessimistic lab results, and hence did not contribute to the assurance calculations; these proportions are shown along the top of the plots for each lab sample size.

When the prevalence of COVID in the target population increases, fewer patients are needed to obtain the adequate sensitivity, and thus there is a decrease in the required sample size for the diagnostic accuracy study. This is consistent across all lab sample sizes, but most prominent for the smaller lab sizes. For example, when $n_c=10$ in Fig. 3 (top plot), the possible sample sizes (stated above) for a prevalence of 0.3 are more than $87\%$ smaller than those for a prevalence of 0.05.

For each lab sample size in the specificity plots (Fig. 4), the opposite relationship between the required sample size and prevalence is observed (compared to sensitivity). Specifically, as the prevalence of COVID increases from 0.05 to 0.3, the proportion of “non-COVID” individuals in the target population decreases, and thus the sample size required to obtain adequate precision around the specificity estimate increases to compensate for this. Moreover, the required sample sizes are much smaller than for the sensitivity case because each sample taken from the target population will contain a larger proportion of non-COVID patients. The proportion of pessimistic samples that are discarded after the initial lab study is higher than in the sensitivity case to reflect the stricter specificity target.

When assuring *both* sensitivity and specificity together in Fig. 5, the sample sizes obtained via the BAM are similar to those for the sensitivity alone case in Fig. 3. However, there are important differences. Most notably, for larger lab sample sizes and higher prevalences, there is an increase in the range of possible sample sizes required. If we had chosen to only assure the sensitivity, assuming that this would be the most stringent target, then there is a risk that the sample size would not have been large enough to assure the specificity. In addition, approximately twice as many samples are rejected for being too pessimistic than in the sensitivity case because they now have to meet the targets on two accuracy measures.

For all cases, similar patterns hold when the target assurance is raised to $90\%$. The main difference is that the required sample sizes are shifted upwards, most notably for the smallest prevalence, and the range of possible sample sizes is generally increased. The minimum and maximum sample sizes required to obtain a sensitivity with the required precision for a target assurance of 80% and 90% are shown in Table 5. Some scenarios return a minimum sample size of 10, i.e. the initial value used in the sample size search. This means that, for some of the lab studies in these scenarios, the desired assurance has already been attained. This is more common for the larger lab sizes because the resulting prior distributions tend to have smaller variances, decreasing the credible interval width.

**Table 5:** (Min, max) sample sizes required to achieve the desired precision for sensitivity. *N.B. The initial sample size is set to 10*

		Number with COVID in lab study, $n_c$				
COVID prevalence	Target assurance	10	20	30	40	50
0.05	0.8	(783, 1630)	(66, 1393)	(10, 1080)	(10, 745)	(10, 403)
	0.9	(1142, 2233)	(196, 1901)	(10, 1476)	(10, 1022)	(10, 558)
0.1	0.8	(317, 605)	(22, 511)	(10, 396)	(10, 275)	(10, 152)
	0.9	(416, 708)	(90, 596)	(10, 463)	(10, 324)	(10, 182)
0.2	0.8	(152, 278)	(10, 232)	(10, 180)	(10, 125)	(10, 70)
	0.9	(194, 307)	(44, 256)	(10, 200)	(10, 141)	(10, 80)
0.3	0.8	(101, 182)	(10, 151)	(10, 117)	(10, 82)	(10, 45)
	0.9	(128, 198)	(29, 165)	(10, 128)	(10, 90)	(10, 51)

Common to all cases is that as the size of the lab study increases, the proportion of samples that are rejected for being too pessimistic decreases and the required sample size, on average, also decreases for each prevalence; markedly so for the smaller prevalences when assuring sensitivity. For example, taking the first plot in Fig. 3 (sensitivity), if the prevalence is 0.05 and $n_c$ increases from 20 to 30 (equivalent to the *total* lab size increasing from 40 to 60), then the median size of the diagnostic accuracy study reduces from 1080 to 858. In this case, for an extra 20 samples in the lab study, the median size of the diagnostic accuracy study could be reduced by 184 to achieve the desired precision around the sensitivity estimate.

### Comparison to alternative methods

We now compare the sample sizes obtained via the BAM to those from the alternative methods described in the “Alternative methods” section, namely, Clopper-Pearson (CP), Agresti-Coull (AC), Wald, Jeffreys and Wilson.

**Fig. 6:**
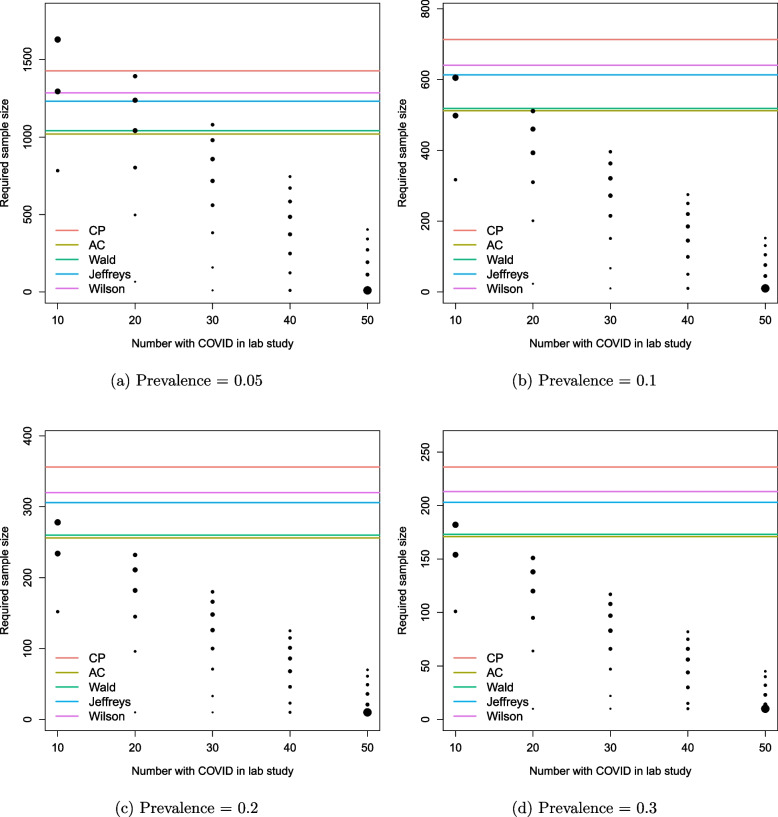
Sample sizes obtained via the BAM with pessimistic lab data excluded (black dots) vs. alternative methods (coloured horizontal lines) for *sensitivity *when assurance/power is 0.8

When implementing the alternative methods, we initially assume that the true values of sensitivity or specificity are known. This gives rise to the sample sizes displayed on Fig. 6 as horizontal lines for comparison with the sample sizes obtained from the BAM (represented by black dots). Each sub-figure corresponds to a different prevalence and shows the range of sample sizes required for a diagnostic accuracy study to achieve the desired precision for the sensitivity with a target power/assurance of 0.8. The analogous plots for specificity are shown in Fig. 11 of the Appendix. Note that, for the BAM, the pessimistic lab data has been excluded from the sample size calculations (as described in the “Simulation study: application to COVID-19” section). In contrast, Fig. 12 in the Appendix shows the corresponding plots when the pessimistic lab data is *included* in the sample size calculations for the diagnostic accuracy study. We see that, by proceeding with tests that do not look like they will satisfy the desired criteria, we can require very large diagnostic accuracy studies, which are unlikely to return a successful test.

For the smallest prevalence of 0.05 in Fig. 6a, all sample sizes from the BAM lie below the sample sizes obtained from each alternative method when $n_c = 40$ and 50. For smaller lab sizes, some of the alternative methods yield smaller sample sizes. For example, when $n_c = 20$, the sample size from AC is smaller than $75\%$ of the BAM sample sizes. When $n_c = 10$, each alternative method — except CP — gives smaller sample sizes than the majority returned by the BAM.

As the prevalence increases, so does the proportion of the BAM sample sizes below the alternative sample sizes. For prevalences of 0.1 and above (Fig. 6b–d), AC and Wald give smaller sample sizes than approximately $45\%$ of the BAM sample sizes for $n_c=10$. However, for the other lab sizes considered, all of the BAM sample sizes are smaller than the alternatives.

**Fig. 7:**
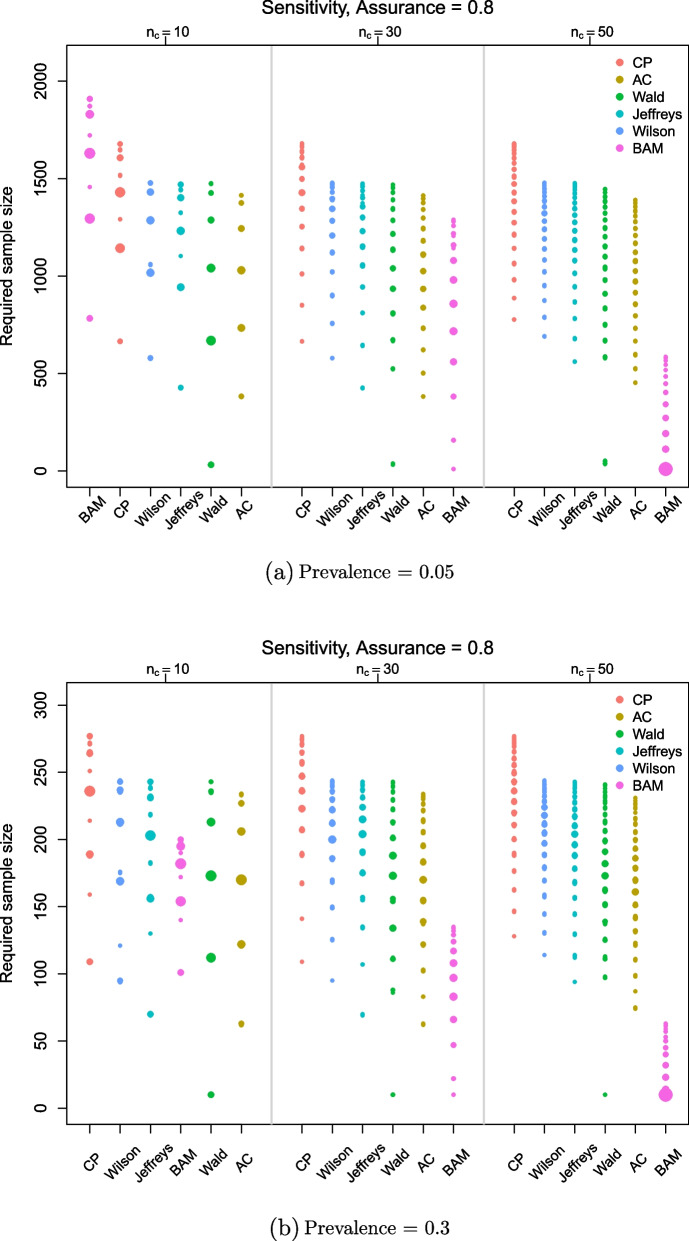
Sample sizes obtained via the BAM (with pessimistic lab data included) vs. alternative methods for *sensitivity *when assurance/power is 0.8

**Fig. 11:**
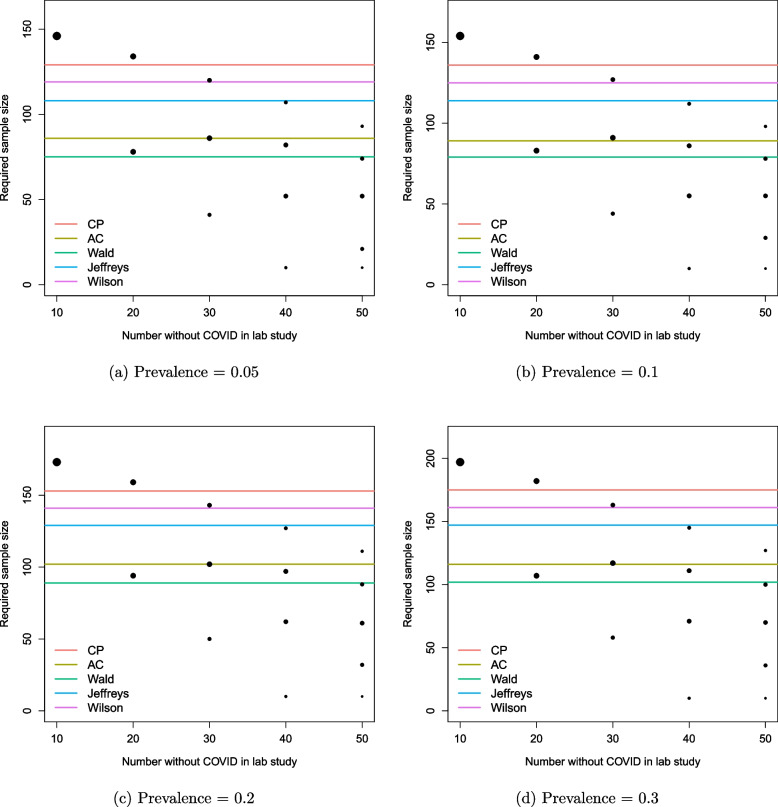
Sample sizes obtained via the BAM with pessimistic lab data *excluded* (black dots) vs. alternative methods (coloured horizontal lines) for *specificity *when assurance/power is 0.8

**Fig. 12:**
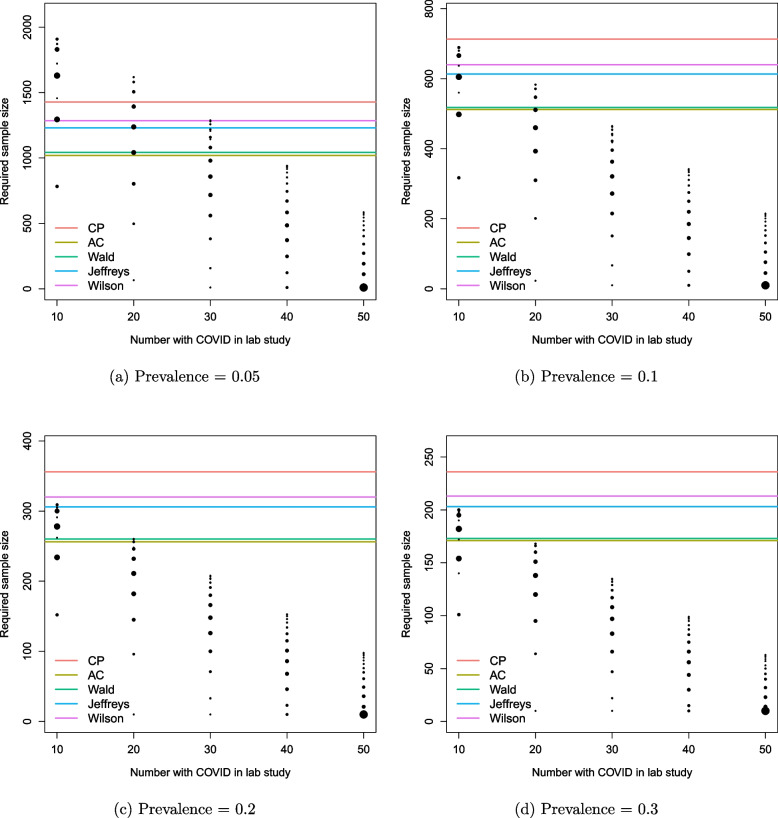
Sample sizes obtained via BAM with pessimistic lab data *included* (black dots) vs. alternative methods (coloured horizontal lines) for *sensitivity *when assurance/power is 0.8

**Fig. 13:**
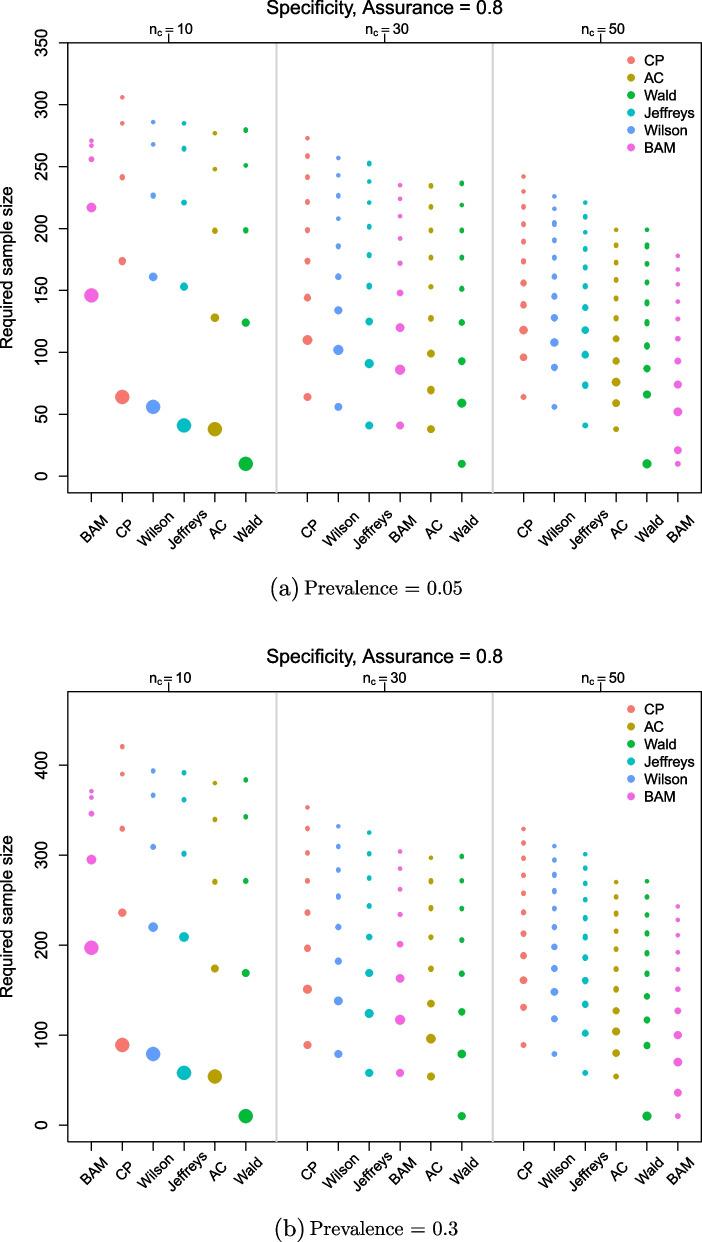
Sample sizes obtained via BAM (with pessimistic lab data *included*) vs. alternative methods for *specificity *when assurance/power is 0.8

**Fig. 14:**
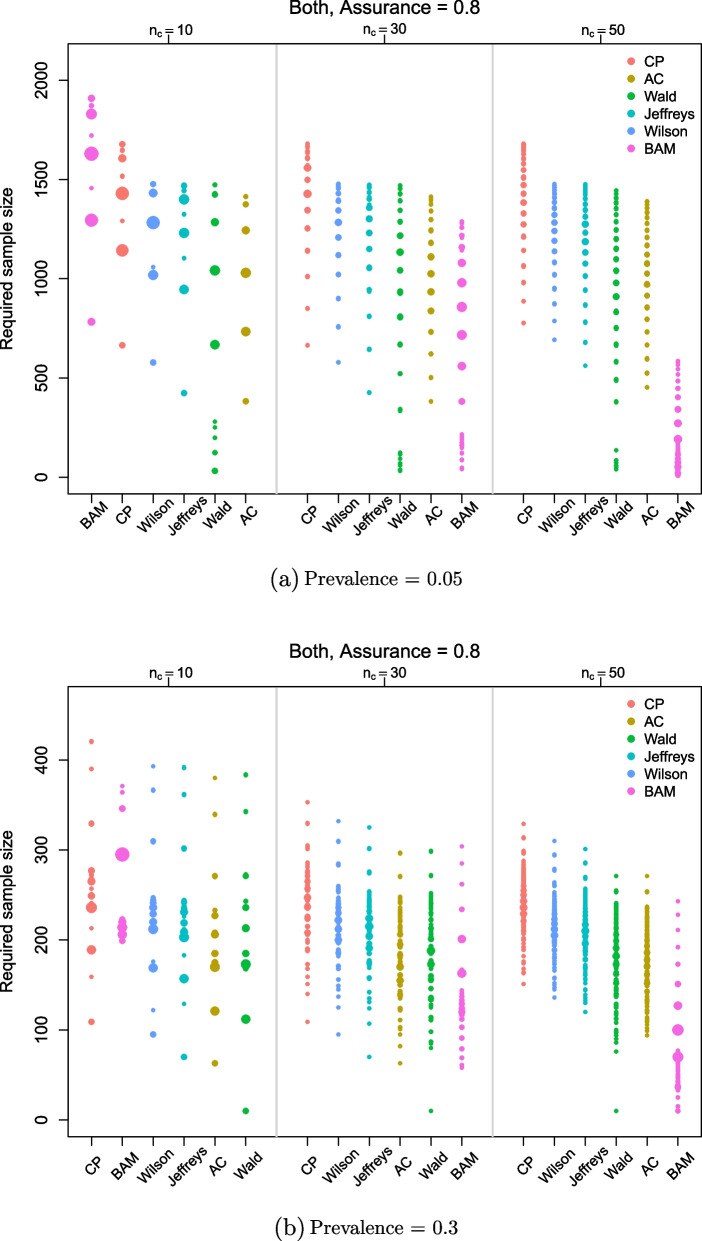
Sample sizes obtained via BAM (with pessimistic lab data *included*) vs. alternative methods for **both sensitivity and specificity** when assurance/power is 0.8

In practice, since the true values of sensitivity and specificity will not be known, we use their maximum likelihood estimates from the lab study in the sample size calculations for the alternative methods. The distribution of sample sizes obtained for sensitivity in a low and high prevalence setting is shown in Fig. 7. Corresponding plots for specificity and both sensitivity and specificity together are provided in Figs. 13 and 14, respectively, of the Appendix. In contrast to previous plots, these results include the pessimistic lab samples.

Within each of the three panels in these plots (corresponding to different lab sample sizes), the methods are ordered according to their median sample size over the $I = 10,000$ simulations. For example, when $\rho _T = 0.05$ and $n_c = n_{\bar{c}} = 10$ in the first panel of Fig. 7a, the alternative methods give smaller median sample sizes than the BAM. When $\rho _T = 0.3$ in Fig. 7b, only AC and Wald have smaller median sample sizes than the BAM. For larger lab study sizes, the BAM has the smallest median sample size across all prevalences.

To determine whether the resulting sensitivity and/or specificity intervals are sufficiently precise, we calculate the proportion of times that they attain the target width. These are summarised in Table 6 for the case when the pessimistic lab data has been excluded from the assurance calculation and the target assurance is 0.8. Analogous results for when the target assurance is 0.9 are shown in Table 7. The results demonstrate that the sample sizes determined using the BAM lead to credible interval widths which successfully attain their target widths with a high probability. Similar results are shown when the pessimistic lab data is instead included.

Table 6 also shows the impact on these results when the data generation process between the lab study population and diagnostic accuracy study population differs. More precisely, we simulate the diagnostic accuracy study population (step 7 of the “Simulation structure” section) from a binomial distribution with values of sensitivity and specificity that are 1%, 5% and 10% smaller than the original values of $\lambda _T=0.8$ and $\theta _T=0.95$ used to generate the lab study samples in step 3 of the “Simulation structure” section. The greater the discrepancy between the two populations, the smaller the proportion of intervals that fall within the target width. A small difference of 1% has little impact on the performance of BAM. A difference of 5% still gives rise to sufficiently narrow credible intervals more often than not. When the difference reaches 10%, we begin to see the success probabilities drop below 50%. A similar trend is shown when the target assurance is raised to 0.9 in Table 7 of the Appendix.

**Table 6:** Proportion of times the posterior credible interval widths attain the target widths when the target assurance is 0.8

	Same				1% smaller				5% smaller				10% smaller			
$\rho _T$	0.05	0.1	0.2	0.3	0.05	0.1	0.2	0.3	0.05	0.1	0.2	0.3	0.05	0.1	0.2	0.3
$n_c$	Sensitivity															
10	0.94	0.84	0.79	0.78	0.94	0.83	0.77	0.75	0.90	0.75	0.67	0.65	0.87	0.65	0.53	0.5
30	0.96	0.88	0.83	0.83	0.95	0.86	0.81	0.80	0.93	0.80	0.73	0.71	0.89	0.69	0.60	0.58
50	0.96	0.93	0.92	0.92	0.96	0.93	0.91	0.92	0.95	0.91	0.89	0.89	0.93	0.88	0.86	0.86
$n_c$	Specificity															
10	1.00	1.00	1.00	1.00	1.00	1.00	1.00	1.00	0.94	0.94	0.93	0.92	0.37	0.37	0.37	0.36
30	0.90	0.90	0.89	0.89	0.86	0.85	0.84	0.84	0.57	0.57	0.56	0.55	0.17	0.17	0.17	0.18
50	0.90	0.90	0.89	0.88	0.86	0.85	0.84	0.84	0.59	0.58	0.57	0.57	0.24	0.23	0.22	0.23
$n_c$	Both															
10	0.94	0.84	0.89	1.00	0.93	0.82	0.87	0.99	0.90	0.74	0.76	0.95	0.87	0.64	0.61	0.50
30	0.96	0.89	0.92	0.94	0.95	0.87	0.90	0.91	0.93	0.80	0.78	0.63	0.88	0.67	0.45	0.20
50	0.93	0.91	0.91	0.89	0.91	0.88	0.87	0.85	0.77	0.69	0.60	0.57	0.58	0.38	0.22	0.22

## Software implementation

To facilitate sample size calculations for diagnostic accuracy studies using the BAM, we have developed an online, interactive application using R Shiny (Version 1.6.0) [7], which is hosted permanently at https://micncltools.shinyapps.io/bayesiansamplesize. This application is designed for non-specialists to be able to implement the BAM.

The implementation of the BAM involves three steps, and is located under the “Assurance” menu. The first step is to define prior distributions for sensitivity and/or specificity. The second step is to define the prior distribution for the prevalence. The third step involves inputting the target sensitivity and/or specificity values and running the sample size calculator.

For ease of use, we have designed the application to allow input of the relevant prior distributions in a number of ways. For the sensitivity, specificity and prevalence priors, the user can directly input the beta distribution parameters.

Alternatively, for the sensitivity $\lambda$ and specificity $\theta$ priors, users can input a $2 \times 2$ table, similar to that provided in Table 2. Given the values in the table, the prior distributions are given by $\lambda \sim \text {Beta}(n_{11}, n_{21})$ and $\theta \sim \text {Beta}(n_{22}, n_{12})$. To ensure the prior distributions are sensible and computationally feasible, the minimum value of each beta distribution parameter has been set to one. For the prevalence, users can instead enter an estimate of the prevalence ($\hat{\rho }_T$) and the sample size from which the estimate came ($n_{\hat{\rho }_T}$). The prior distribution for the prevalence is then given by $\rho _T \sim \text {Beta}(\hat{\rho }_T n_{\hat{\rho }_T},(1-\hat{\rho }_T)n_{\hat{\rho }_T}).$

The final method for specifying the prior distributions is a simple expert elicitation exercise where the user is asked a range of questions to establish (i) an interval for the parameter of interest, (ii) a probability that the parameter lies within the interval, and (iii) a best estimate of the parameter. These values are taken to correspond to (i) a pair of quantile values, (ii) the probability between the two quantile values, and (iii) the median. A beta distribution is then fitted to these values using the least-squares method [23].

Density plots of the fitted prior distributions, the median, a symmetric 95% credible interval, and the prior distribution parameters are provided to the user. Users can choose how to input sensitivity, specificity, and prevalence separately, allowing for different combinations of approaches where the availability of prior information varies.

Following the specification of the prior distributions, the user specifies target values for the assurance calculation, including: target sensitivity and/or specificity interval widths and the required assurance level. Upon calculation, users are provided with the required sample size and an assurance curve showing how the assurance varies with sample size.

## Discussion

In this paper, we have shown how and why novel methods, such as the BAM, can improve efficiency of diagnostic accuracy study designs for COVID diagnostic tests. Overall, we found that the BAM generally outperforms the sample size calculation methods routinely used in practice. However, as we have demonstrated, this will not always be the case. For example, if the prior distributions have larger variances or are centred on different values compared to power calculation inputs, this may result in greater differences between assurance and power calculations. Even when assurance calculations lead to higher sample sizes, the increased granularity in the information from the prior distribution still better reflects the state of knowledge of the diagnostic test than a single point estimate, and thus the larger sample size is more realistic and robust. Increasing the lab sample size can provide a reduction in the total number of samples required in the diagnostic accuracy study. Care should be taken, however, if there are large discrepancies between the samples taken for the lab study and diagnostic accuracy study.

A limitation of the BAM is the assumption that the lab study results are appropriate for use in developing the prior distribution for the subsequent diagnostic accuracy study. It may be the case that the lab results are not expected to reflect those in later studies, such as when there are differences in the type of biological sample used. This is particularly pertinent to clinical areas such as stroke where the analyte is not present at all in samples from healthy controls so the lab samples may have to be spiked. In clinical areas such as cancer, where disease course can be long and complicated, it can also be difficult to acquire lab samples from the correct phase of the disease (e.g. early diagnosis). However, this is less likely to pose an issue in the COVID setting, and for infectious respiratory diseases more generally, where the lab samples are required by regulation to represent a broad range of viral loads, and thus are typically representative of a similar, or slightly broader, population than in hospital or community settings. Nevertheless, assessing prior-data conflict should be an integral part of any Bayesian analysis and if inconsistencies exist, various approaches can be taken to incorporate the information in a more appropriate way. One approach is to use a power prior [18], which involves raising the prior distribution based on the lab study results to some power. This increases the variance of the distribution to reflect a greater level of uncertainty about how the lab and diagnostic accuracy studies differ, but keeps it centred on the same value to reflect the best available knowledge. Another approach is to use commensurate priors [16] or hierarchical models which borrow information between groups (development stages) based on the correlation between results in the different groups.

Typically, in diagnostic accuracy studies, the data is only analysed at the end of the study. Future work could implement the BAM in a group sequential framework so that the data can be monitored sequentially and used to update the posterior credible intervals adaptively at interim analyses. This would allow the study to be stopped early for success or futility based on pre-defined stopping rules. Stopping the study early for success would allow dissemination of the findings and deployment of the test earlier. Stopping early for futility would prevent wasting resources and allow attention to be turned to other competing tests. This could further improve the efficiency of the procedure, which may be very important in a public health emergency like COVID where rapid response is key.

The fact that standard statistical software does not have the embedded functions to implement novel methods such as the BAM is a major barrier to its widespread use across diagnostic study designs. However, the user-friendly interactive web application developed alongside this work can encourage increased uptake and ameliorate reluctance to try unfamiliar methods.

## Conclusions

The BAM presents a Bayesian method of sample size calculation which incorporates prior information from previous studies. Using the case study of rapid diagnostic tests for COVID-19, we have illustrated how the BAM can be applied to determine the required sample size for a diagnostic accuracy study investigating sensitivity and specificity simultaneously or separately.

Applying this approach can often result in smaller sample sizes than those produced using conventional methods. Increasing the size of the lab study can further reduce the required sample size for future diagnostic accuracy studies. This suggests that investing more time and effort in the lab study, to ensure there are sufficient samples available, can bring worthwhile gains. Therefore, the trade-off between lab study sample size and subsequent diagnostic accuracy study sample size is an important consideration.

This work has focused on the application of BAM to the COVID-19 pandemic setting, but the conclusions of this study are also important for the future development of tests in other areas.

## Figures and Tables

**Fig. 8:**
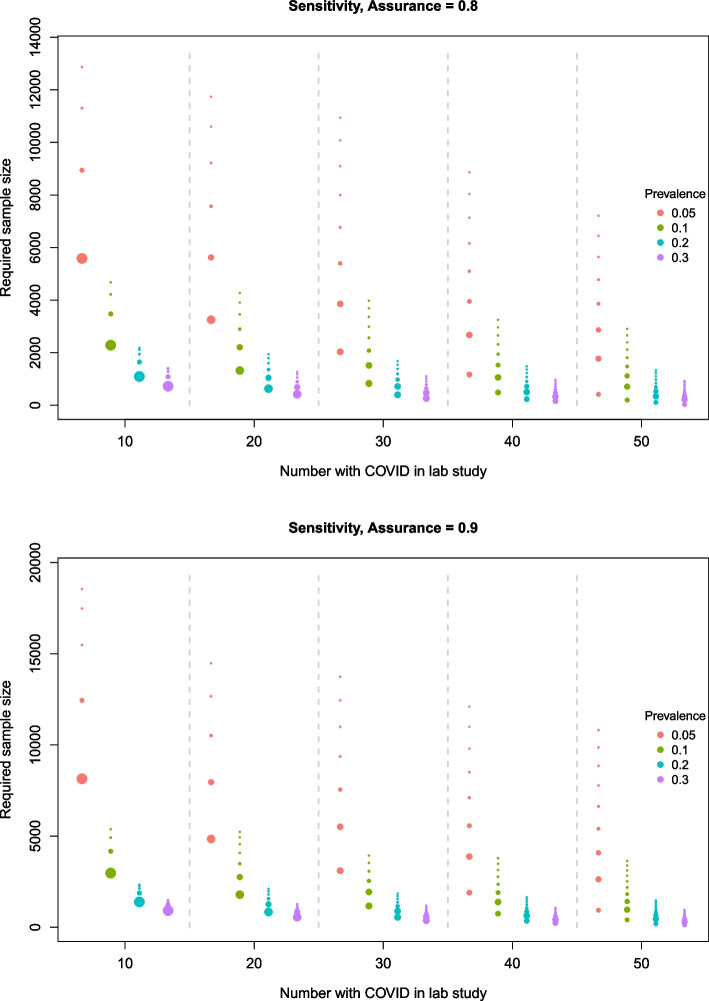
Required sample sizes for different lab sample sizes and prevalences based on the BAM for *sensitivity*. The top and bottom plots correspond to a target assurance of 0.8 and 0.9, respectively

**Fig. 9:**
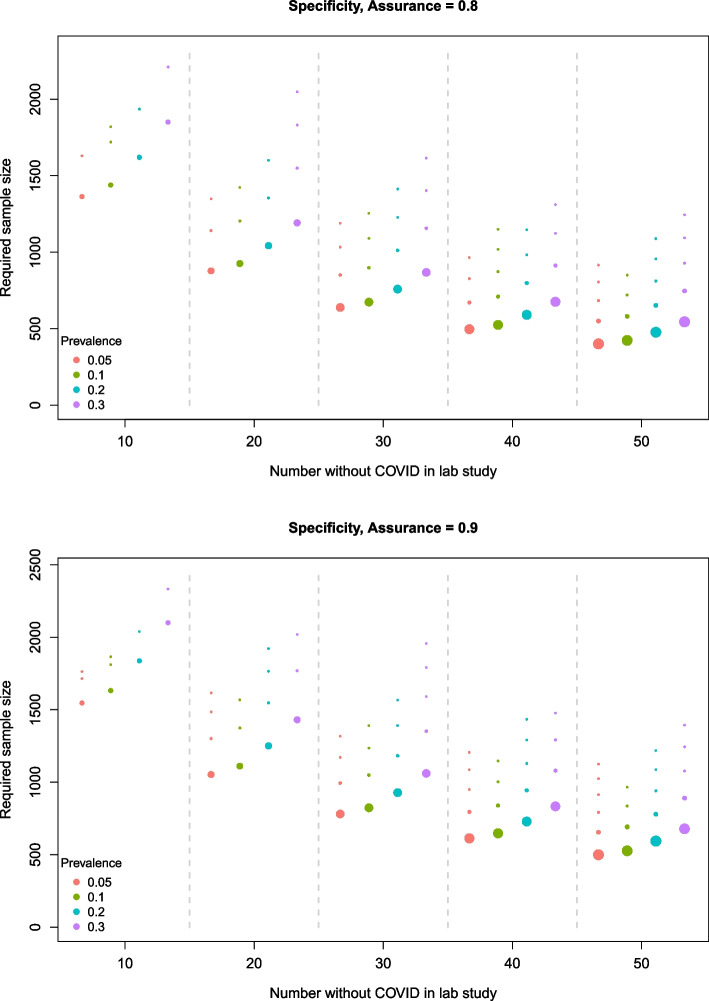
Required sample sizes for different lab sample sizes and prevalences based on the BAM for *specificity*. The top and bottom plots correspond to a target assurance of 0.8 and 0.9, respectively

**Fig. 10:**
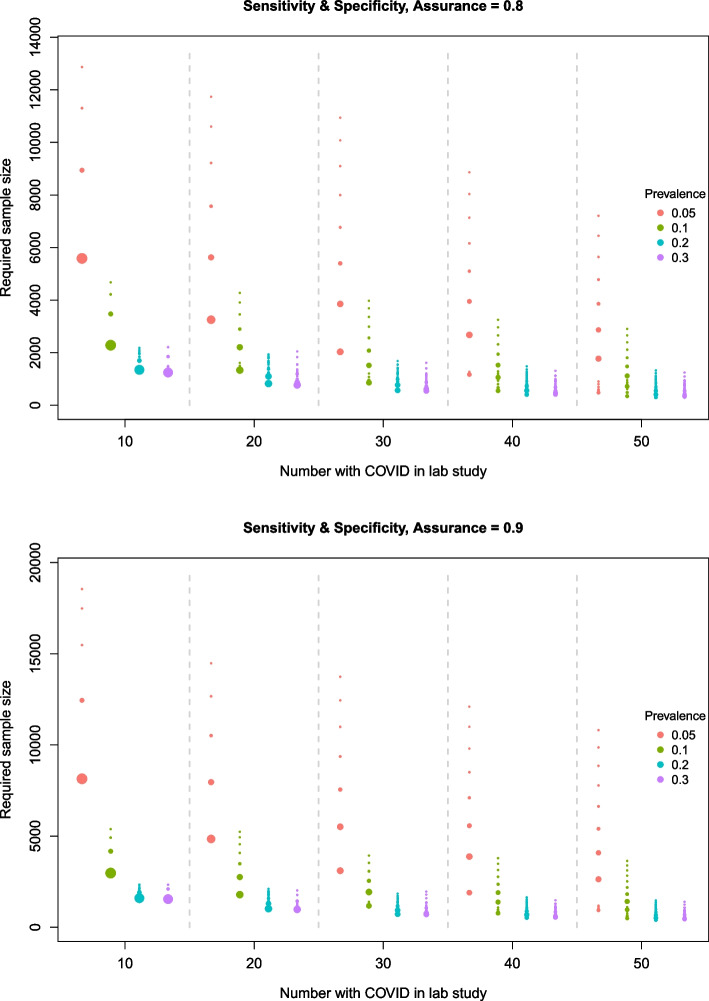
Required sample sizes for different lab sample sizes and prevalences based on the BAM for *sensitivity and specificity* together. The top and bottom plots correspond to a target assurance of 0.8 and 0.9, respectively

## Data Availability

The R code to implement the Bayesian assurance method (BAM) and power-based methods is available from the corresponding author upon request. An online web application which performs the sample size calculations using the BAM is available at: https://micncltools.shinyapps.io/bayesiansamplesize/. The R code used to generate the simulated data used for the analysis is available on request.

## Appendix

### Performance of the BAM for the desirable TPPs

Figures 8, 9 and 10 show the required sample sizes corresponding to the desirable TPPs (provided in Table 1).

### BAM vs. alternatives

Figure 11 is the analogue to Fig. 6 in the main paper but for specificity.

Figure 12 includes the full set of lab results (including the pessimistic samples).

### Performance of the BAM: interval widths

**Table 7:** Proportion of times the posterior credible interval widths attain the target widths when the target assurance is 0.9

	Same				1% smaller				5% smaller				10% smaller			
$\rho _T$	0.05	0.1	0.2	0.3	0.05	0.1	0.2	0.3	0.05	0.1	0.2	0.3	0.05	0.1	0.2	0.3
$n_c$	Sensitivity															
10	1.00	0.95	0.92	0.91	1.00	0.94	0.90	0.89	0.99	0.91	0.84	0.82	0.98	0.86	0.74	0.71
30	0.99	0.96	0.93	0.92	0.99	0.95	0.92	0.90	0.98	0.91	0.86	0.84	0.97	0.85	0.77	0.74
50	0.99	0.97	0.96	0.96	0.98	0.96	0.95	0.96	0.98	0.95	0.94	0.94	0.97	0.93	0.92	0.92
$n_c$	Specificity															
10	1.00	1.00	1.00	1.00	1.00	1.00	1.00	1.00	1.00	1.00	1.00	1.00	0.94	0.94	0.93	0.93
30	0.96	0.96	0.96	0.96	0.94	0.94	0.94	0.94	0.75	0.76	0.76	0.75	0.38	0.38	0.38	0.38
50	0.96	0.96	0.96	0.96	0.94	0.94	0.93	0.93	0.75	0.75	0.75	0.74	0.37	0.37	0.37	0.37
$n_c$	Both															
10	1.00	0.95	0.97	1.00	1.00	0.94	0.96	1.00	0.99	0.90	0.92	1.00	0.98	0.85	0.84	0.94
30	0.99	0.96	0.98	0.99	0.99	0.95	0.97	0.97	0.98	0.92	0.91	0.82	0.96	0.85	0.70	0.41
50	0.98	0.97	0.96	0.96	0.96	0.95	0.95	0.94	0.88	0.84	0.77	0.74	0.70	0.55	0.37	0.36
